# Tumor‐driven SRS VMAT planning: Regression models for intermediate and low dose spillage

**DOI:** 10.1002/acm2.70184

**Published:** 2025-07-31

**Authors:** Meysam Tavakoli, Shada Wadi‐Ramahi, Sarah Ashmeg, Ron Lalonde, Zaid Siddiqui

**Affiliations:** ^1^ Department of Radiation Oncology and Winship Cancer Institute Emory University Atlanta Georgia USA; ^2^ Department of Radiation Oncology University of Pittsburgh School of Medicine and UPMC Hillman Cancer Centre Pittsburgh Pennsylvania USA; ^3^ Department of Radiation Oncology Baylor College of Medicine Houston Texas USA

**Keywords:** low and intermediate dose, multi‐metastatic, regression models, single isocenter, SRS

## Abstract

**Purpose:**

Stereotactic radiosurgery (SRS) for brain metastases using volumetric modulated arc therapy (VMAT) is increasingly utilized. While high‐dose conformity guidelines relative to tumor volume exist, recommendations for intermediate and low‐dose regions remain undefined. This study explores tumor‐specific characteristics and new dosimetric parameters to develop regression models for standardizing intracranial SRS planning.

**Materials and Method:**

We introduce two dosimetric quantities: R_6Gy_, the 6 Gy cloud volume ratio to the PTV, and %D_1cm_, the maximum dose at 1 cm from the PTV relative to the prescribed dose. These, alongside R_50%_ and the volume of normal brain receiving 12 Gy (V_12Gy_), were analyzed retrospectively in 290 VMAT SRS plans from 151 patients treated between January 2021 and September 2023. The data were stratified into single‐ and three‐ fraction arms. Statistical tests, including Spearman's rank correlation, and Normalized Mutual Information (NMI) evaluated relationships between dosimetric parameters, number of metastases (*n*), and total PTV volume, PTV_Total_. Significant correlations were modeled using regression analysis.

**Results:**

Strong correlations were found between PTV_Total_ and all dosimetric metrics in the single‐fraction arm; weaker but significant correlations were noted in the three‐fraction arm. Power‐law regression best described R_50%_ and R_6Gy_, while linear regressions best described %D_1cm_ and V_12Gy_. Moderate monotonic correlations were observed between n and the dosimetric metrics.

**Conclusion:**

This study proposes regression‐based models for predicting dose spill based on tumor burden, total PTV volume and number of targets. These models provide a framework for model‐based SRS planning, offering clinical physicists patient‐specific guidance to improve consistency, optimize plan quality, and support future standardization efforts.

## 1 INTRODUCTION

Volumetric modulated arc therapy (VMAT) is increasingly used for stereotactic radiosurgery (SRS), with recent efforts focused on achieving plan quality comparable to dedicated systems like Gamma Knife.^1^, ^2^, ^3^, ^4^, ^5^, ^6^, ^7^, ^8^, ^9^, ^10^, ^11^, ^12^, ^13^, ^14^ While plan evaluation metrics such as conformity index (CI), gradient index and dose‐volume limits for healthy brain are well established in Gamma Knife literature,^15^, ^16^, ^17^, ^18^, ^19^, ^20^, ^21^, ^22^ VMAT planning poses unique challenges. Its dependence on MLC characteristics and beam modulation can make it more difficult to achieve consistent conformity and dose fall‐off, especially in multi‐target cases.^23^, ^24^, ^25^, ^26^, ^27^

Several recent studies have attempted to correlate tumor characteristics ‐ such as the number of metastases and total volume of PTV ‐ with dosimetric performance. Desai et al.^28^ proposed the *R*_50%_ metric for intermediate dose spill and found a power‐law relationship with target diameter. De Camargo et al.^29^ reported correlations between plan quality and target volume but noted weak correlation with the number of targets. They also found dependence on plan quality and the location of the targets relative to the isocenter. Other studies contradicted these findings or used mixed planning techniques (VMAT and DCA), limiting generalizability.^30^, ^31^ Others compared plans for multi‐target brain SRS for different linac platforms but the literature is not cohesive due to variability on how the comparison is made.^32^, ^33^, ^34^, ^35^ Collectively, these works highlight the lack of standardized benchmarks for intermediate and low‐dose spillage in VMAT SRS planning, particularly for multi‐target cases where plan complexity and dose bridging present significant challenges.

In this study, we aim to address these gaps by performing a comprehensive correlation analysis between tumor‐specific characteristics – namely total PTV and target count – and key dosimetric quantities in a large dataset of clinically delivered, single‐isocenter, non‐coplanar VMAT SRS plans. We introduce two novel low‐dose metrics: R_6Gy_, the ratio of the 6Gy cloud to PTV_Total_ volume, and %D_1cm_, a linear dose gradient metric at 1cm from PTV_Total_. While our analysis includes both single‐ and multiple‐target cases, our focus is on developing tumor‐specific models that support clinical decision‐making and standardization of planning.

## 2 METHODS AND MATERIAL

### 2.1 Patient selection

In this study a retrospective analysis of the patients treated at our institution was done. Institution Review Board approval, STUDY20070273, was obtained to access the database of patients treated for brain metastases between January 2021 and September 2023. The database included 1900 patients whose plans were created in Eclipse external beam planning system (Varian Medical Systems, Siemens Healthineers, Palo Alto, CA), using Anisotropic analytical algorithm (AAA) for plan calculation with a dose grid of 1 mm. Plans were created using HyperArc ™, a non‐coplanar VMAT technique with 3 or 4 arc beams. Treatment was carried out on a Varian TrueBeam STX® linac with high‐definition MLC (HD120 MLC) using 6FFF beam with a nominal 1400 MU/min dose rate.

From this database, we selected the first 151 patients who had a single dose prescription to the PTV, resulting in a total number of 295 SRS plans. Five plans were removed due to inconsistency in fractionations, these plans were prescribed two and five fractions treatment. The remaining 290 plans were divided into two arms, single fraction and three fractions. Table 1 summarizes the characteristics of the plans.

**TABLE 1:** Characteristics of the plans used in this study.

	Single fraction	Three fractions
Number of plans	234	56
Total number of lesions	734	144
Max number of lesions in any single plan	23	14
Min number of lesions in any one plan (number of plans).	1 (39 plans)	1 (25 plans)
Max PTV_Total_ volume in any one plan, cc	45.2	65.8
Min PTV_Total_ volume in any one plan, cc	0.1	0.1
Median Prescribed dose, cGy	1800	2400
Range of prescribed dose, cGy	1200 ‐ 2400	1800 ‐ 2700
Average CI + S.D.	1.16 ± 0.17	1.07 ± 0.09
Median CI	1.11	1.06
Median MF	3.30	3.54

Abbreviations: CI, conformity index; MF, modulation factor described as the ratio of the monitor units to the prescribed fractional dose; S.D., Standard deviation.

Notably, single target cases were present in both arms, comprising 38 of the 234 single‐fraction plans, and 25 of the 56 in the three‐fraction plans. All plans were the clinical plans used for patients’ treatments. None of the plans were re‐optimized or otherwise changed for this study. In our practice a 1 mm expansion is used for each brain metastasis to create the PTV. Tumor characteristics considered in this study are number of targets, n, and cumulative PTV volume (PTV_Total_).

### 2.2 Dosimetric quantities

For this work we are primarily interested in correlating targets’ characteristics; total volume and number of metastases (*PTV_Total_*, *n*), to intermediate and low‐dose spillage and to volume of brain (minus GTV) receiving 12Gy, V_12Gy_.

For the intermediate‐dose gradient, *R*_50%_, we used the concept introduced by Desai et al.^28^ defined as the ratio of 50% dose cloud to the PTV_Total_. In contrast to Desai et al, we measured *R*_50%_ directly from each plan instead of calculating it from the gradient index (GI). The volume of 50% dose cloud was converted to a structure and R_50%_ represents the ratio of 50% volume to that of PTV_Total_.

We are also introducing two additional dosimetric quantities for the low‐dose spillage; *R*_6_ *_Gy_* defined as the ratio of the 6Gy cloud volume to the *PTV_Total_*, and %*D*_1_ *_cm_*, defined as the maximum dose measured at 1.0 cm away from *PTV_Total_* in any direction relative to the prescription dose. %D_1cm_ is intended as a linear‐dose falloff metric that supplements volumetric metrics. The simplicity of this linear metric, especially in multiple metastatic cases, is to give the planner a quick one‐dimensional check of the dose spillage beyond the PTV. It is a useful tool to identify outliers in plan quality and for comparison of various plans. Dose gradients in intermediate and low dose regions can be better understood and controlled when using a volume‐based quantities, R_50%_ and R_6Gy_, as well as a linear quantity %D_1cm_. Finding %*D*_1_ *_cm_* required multiple steps. First all PTV targets were combined into one single target *PTV_Total_*, then the ring function in the planning system was used to create a ring structure with the inner diameter at 1.0 cm away from *PTV_Total_*. The maximum dose to the ring structure is then taken as a percentage of the prescribed dose.

In both arms, there were multiple plans (60 and 28 in the single‐ and three‐ fractions, respectively) with explicit dose prescription to the GTV. For those plans, and to keep the definition of R_50%_ and %D_1cm_ consistent, we used the prescribed dose to the PTV as the reference dose.

For assessing dose to normal brain tissue, we use *V*_12_ *_Gy_* defined as the volume of normal brain (brain – GTV_Total_) receiving 12 Gy.

### 2.3 Statistical analysis

We used Spearman's rank correlation test to evaluate potential monotonic relationships between *PTV_Total_* and the various dosimetric parameters, *R*_50%_, *R*_6Gy_, *V*_12Gy_. Spearman's test is a non‐ parametric method that assesses the strength and direction of monotonic associations between two continuous or ordinal variables. It does not assume a normal distribution and is robust to outliers, making it particularly useful for clinical data. Prior to applying the test, we visually assessed scatter plots to verify that the relationship between variables appeared monotonic, which is a key assumption of the Spearman method.

The number of targets, *n*, is a discrete variable and assessing its relationship with continuous dosimetric metrics required a combination of statistical approaches. We applied both Spearman rank test and the Normalized Mutual Information (NMI) test. NMI is well‐suited for detecting nonlinear or complex relationships and can handle continuous, discrete, or mixed data types.

Spearman's correlation tests were performed using GraphPad Prsim 6, using a significance threshold of *p* = 0.05. NMI calculations were performed in Python using the scikit‐learn library.

## 3 RESULTS

### 3.1 Dependance of the dosimetrical parameters on *PTV_Total_*

For the single fraction arm, the Spearman test showed a strong correlation that is statistically significant for all the dosimetrical quantities. In the three‐fraction arm, strong and significant correlation was found for V_12Gy_ and %D_1cm_ with a weak correlation that is marginally significant for R_50%_ and R_6Gy_. Table 2 summarizes these details.

**TABLE 2:** Correlation between PTV_Total_ and dosimetric parameters.

	Single fraction arm			Three‐fractions arm		
	Spearman	p‐value	Correlation and significance?	Spearman	p‐value	Correlation and significance?
R_50%_	−0.68	6.72 × 10^−8^	Strong and significant correlation	−*0.371*	*0.00478*	Weak, marginal significance
R_6Gy_	−0.672	2.92 × 10^−32^	Strong and significant correlation	−*0.282*	*0.036*	Weak, marginal significance
%D_1cm_	0.61	6.15 × 10^−7^	Strong and significant correlation	0.592	1.51 × 10^−6^	Strong, significant correlation
V_12Gy_	0.895	1.48 × 10^−20^	Strong and significant correlation	0.844	2.94 × 10^−16^	Strong, significant correlation

Both R_50%_ and R_6Gy_ have negative Spearman correlation indicating that they are inversely related to PTV_Total_. We found that the power law was the best fit for R_50%_ and R_6Gy,_ with the relationship given as

(1) $$\begin{array}{cc} \mathrm{Single}\;\mathrm{fraction}\;\mathrm{arm}: & R_{50\%}=6.769\mathrm{PT}V^{-0.245}\\ \; & R^{2}=0.2339 \end{array}$$

(2) $$\begin{array}{c} R_{6\mathrm{Gy}}=15.164\mathrm{PT}V^{-0.336}\\ R^{2}=0.1508 \end{array}$$

(3) $$\begin{array}{cc} \mathrm{Three}\;\mathrm{fractions}\;\mathrm{arm}: & R_{50\%}=5.1165\mathrm{PT}V^{-0.167}\\ \; & R^{2}=0.4469 \end{array}$$

(4) $$\begin{array}{c} R_{6\mathrm{Gy}}=16.205\mathrm{PT}V^{-0.181}\\ R^{2}=0.2957 \end{array}$$

**FIGURE 1:**
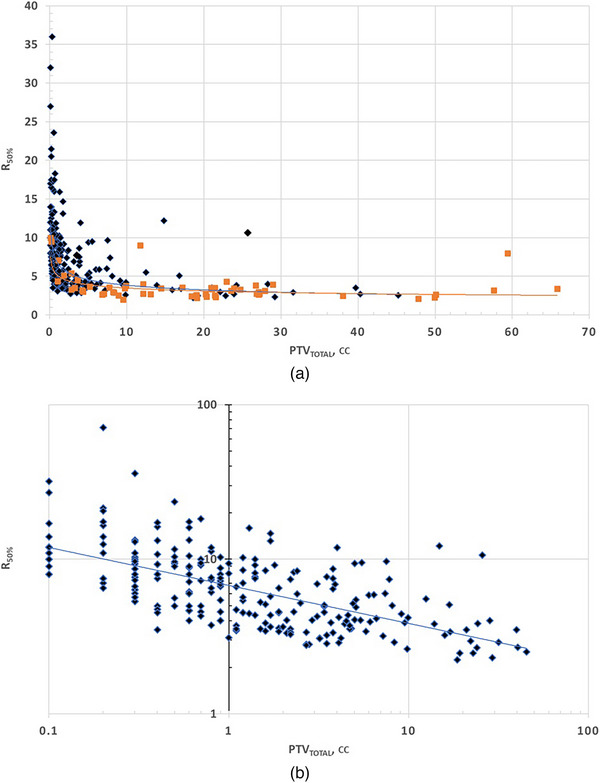
R_50%_ versus PTV_Total_ in (a) a linear graph for both arms. The single fraction arm has diamond data points, and the three fractions arm has square points, (b) a log‐log graph for the single fraction arm, shown for clarity of regression.

Both intermediate, R_50%_, and low dose, R_6Gy_, quantities have similar plots. In Figure 1, we chose to present R_50%_.

This negative monotonic relationship implies that as the total tumor volume increases, the relative intermediate (R_50%_) and low‐dose spill (R_6Gy_) consistently decrease. Clinically, these findings suggest that dosimetric spill metrics such as %D1cm and V12Gy scale predictably with target volume, enabling planners to establish practical benchmarks for acceptable dose spillage – particularly in high‐burden cases. While larger target volumes may appear to demonstrate more favorable conformity metrics, this is partly influenced by the relative impact of the denominator in ratio‐based metrics, for example, R_50%_, which can overstate dose spill in very small targets. The positive Spearman correlation for %D_1cm_ and V_12Gy_ indicates a monotonic increase with PTV_Total_, and visual inspection of the scatter plots showed that a linear regression provided the best overall fit. These trends can support more objective evaluation criteria in VMAT SRS planning for varying tumor burdens.

The relationship is described by:

Single fraction arm:

(5) $$\begin{array}{c} R_{12\mathrm{Gy}}=1.2\mathrm{PTV}+3.23\\ R^{2}=0.6529 \end{array}$$

(6) $$\begin{array}{c} \%D_{1\mathrm{cm}}=0.7141+30.392\\ R^{2}=0.2584 \end{array}$$

Three fractions arm:

(7) $$\begin{array}{c} V_{12\mathrm{Gy}}=1.495\mathrm{PTV}+11.78\\ R^{2}=0.685 \end{array}$$

(8) $$\begin{array}{c} \%D_{1\mathrm{cm}}=0.4734\mathrm{PTV}+31.79\\ R^{2}=0.3387 \end{array}$$

**FIGURE 2:**
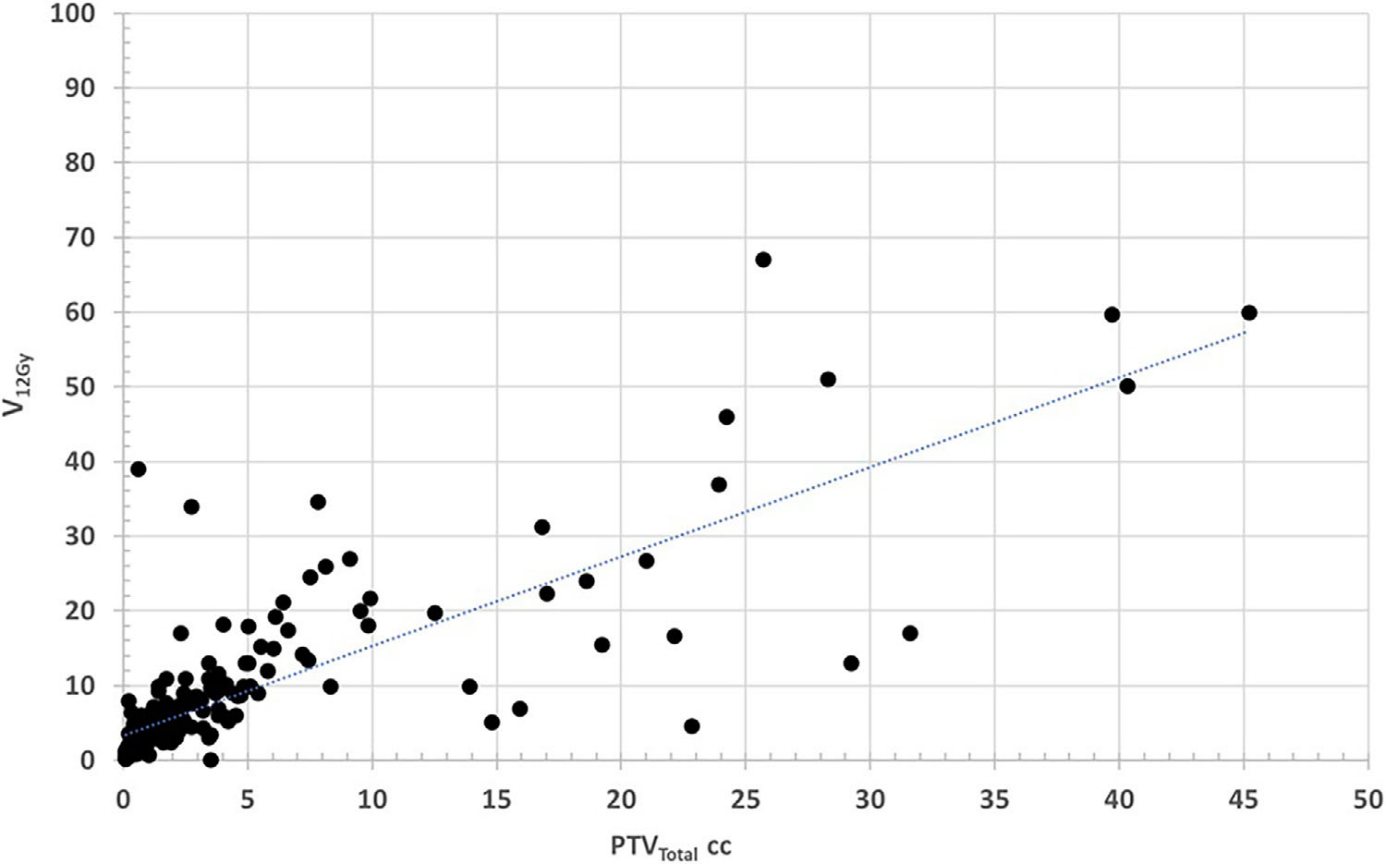
Scatter plot of V_12Gy_ versus PTV_Total_ for the single fraction arm showing the linear regression.

**FIGURE 3:**
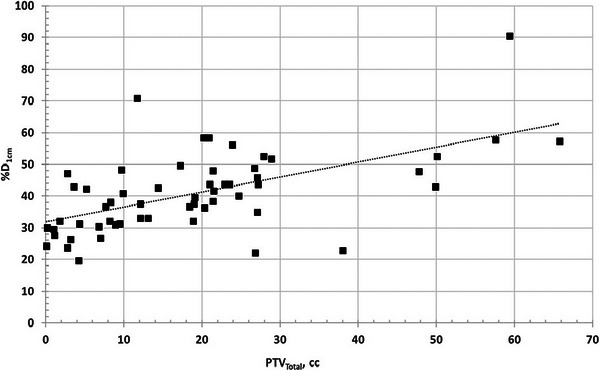
Scatter plot of %D_1cm_ versus PTV_Total_ for three fractions arm.

Figures 2 and 3 both illustrate the linear relationship between PTV_Total_ and V_12Gy_ %D_1cm_, respectively, both for the single fraction arm.

The positive monotonic trend for %D_1cm_ and V_12Gy_ means that as tumor volume increases, so does the dose spill into surrounding tissue. This is clinically significant, as it reflects the challenge of achieving a tight dose fall‐off in large or complex volumes. These trends may assist in setting volume‐specific expectations during plan review.

### 3.2 Dependence of the dosimetric parameters on the total number of targets, *n*

**FIGURE 4:**
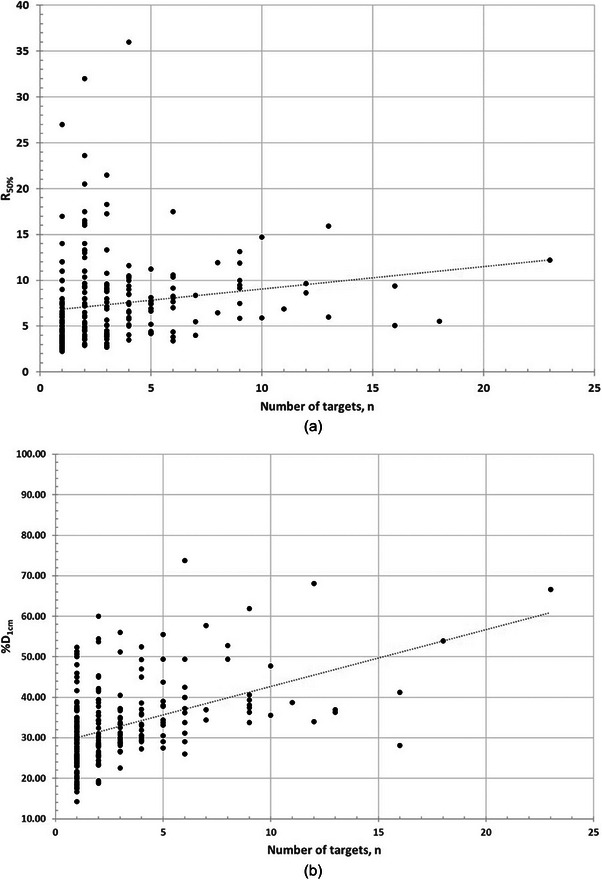
Scatter plot of (a) R_50%_ and (b) %D_1cm_ for the single‐fraction arm.

Scatter plots of the number of targets, n, versus the dosimetric quantities reveal a many‐to‐one relationship, as illustrated in Figure 4. While we chose to illustrate the relation of R_50%_ and %D_1cm_ versus n, for the single fraction arm, the other quantities in both arms show similar behavior. A many‐to‐one relation indicates that patients with the same number of targets can exhibit a wide range of dosimetric values, suggesting the presence of a complex, non‐deterministic relationship between the target count and the dose metrics. In Figure 4, a linear regression line is plotted to assess, visually, if a monotonic relationship can be observed before applying Spearman rank correlation. Table 3 presents the results of the Spearman rank correlation and NMI.

**TABLE 3:** Spearman Correlation and Normalized Mutual Information (NMI) tests between the dosimetric parameters and n.

	Single fraction arm			Three fractions arm		
	Spearman coefficient	Spearman p‐value	NMI	Spearman coefficient	Spearman p‐value	NMI
R_50%_	0.375	2.81 × 10^−9^	0.487	0.398	0.0024	0.586
R_6Gy_	0.302	2.34 × 10^−6^	0.498	0.591	1.61 × 10^−6^	0.586

%D_1cm_	0.462	7.80 × 10^−14^	0.447	0.431	0.00092	0.540
V_12Gy_	0.479	7.51 × 10^−15^	0.396	0.395	0.0026	0.576

In the single‐fraction arm the Spearman test showed a moderate to strong positive correlation, consistent with the NMI findings, which indicated a moderate degree of statistical dependency. In the three‐fractions arm, Spearman test was still showing a moderate correlation and NMI continued to indicate non‐trivial dependency, albeit slightly weaker than in the single‐fraction arm.

## 4 DISCUSSION

The purpose of this study was to present two novel low‐dose spill metrics and evaluate their relationship with established parameters to develop regression models aimed at standardizing intracranial SRS planning. We found that all the studied dosimetric quantities strongly correlated to PTV_Total_ with high significance in the single fraction arm and weak to strong correlation in the three fractions arm with mixed significance. Our power‐law regression fit for R_50%_ is similar to the result found by Desai et al.^28^. Whereas they calculated R_50%_ from other parameters and used it without testing correlation, we verified a strong correlation using Spearman test. In addition, our dataset extends to much smaller targets than Desai et al.^28^ thus showing that the relation holds for smaller volumes. Hence establishing R_50%_ as a useful guidance for the intermediate dose spillage. A similar power‐law fit was found for our newly proposed low‐dose spillage, R_6Gy_, making it another useful guidance for low‐dose spillage.

Both V_12Gy_ and the newly proposed linear gradient at 1cm, %D_1cm_, showed strong positive correlation with a linear regression relationship to PTV_Total_ in both arms of this study. The low value of the regression fit, R^2^, does not negate the strong result of Spearman correlation. We interpret these R^2^ values as either representing statistical noise within this limited clinical dataset or to indicating the presence of additional influencing factors. One such factor is the geometric location of the targets relative to critical structures. When targets are adjacent to sensitive brain regions, planners often prioritize OAR sparing, leading to increased dose spillage outside the target. Additionally, the spatial proximity of the targets to one another introduces further complexities. When multiple metastases are closely spaced, MLC systems, especially those with larger leaf widths, may not provide sufficient resolution to distinguish between neighboring targets. This limitation can lead to dose bridging, degrading plan quality and increasing low‐ and intermediate‐dose spill. These geometric factors are not captured in our current model but are important considerations for future predictive frameworks.

The correlation with the number of metastases was assessed using both Spearman's rank correlation and Normalized Mutual Information (NMI). Each test offers a different perspective–‐ Spearman evaluates the strength of the monotonic trend, while NMI captures broader statistical dependencies, including non‐monotonic relationships. When interpreted together, the results indicate that there is, at best, a moderate correlation between the number of targets and the quantities studied without a clear single regression equation.

This complexity likely reflects the influence of additional factors, as previously suggested by de Camargo.^29^ Such factors may include the spatial distribution of the targets (clustering vs. separation), proximity to the isocenter, dose fall‐off constraints near OARs, and specific beam and MLC geometries – all of which can influence dosimetric parameters in non‐linear ways. It is important to interpret these relationships in clinically meaningful terms. A monotonic relationship indicates a consistent directional trend between tumor burden and a given dosimetric parameter – either always increasing or always decreasing. This scalability with tumor volume enables predictability and allows modelling for the intermediate and low‐dose metrics, as well as V_12Gy_. These trends can help planners set realistic expectations, distinguish outlier plans, and optimize dose gradients appropriately for each case.

In their efforts to benchmark the practice of stereotactic radiosurgery across the United Kingdom, Eaton et al.^36^ found variations in clinical practice and priorities of planning outcomes. All participants favored PTV coverage and high‐dose conformity, however there were larger variations in gradient indices and low‐dose spillage. They concluded their work by calling for standardization of practice for linac‐based SRS treatment. We believe our regression‐based models represent a step toward that goal by offering practical, tumor‐specific benchmarks for plan evaluation.

While all plans in this retrospective analysis were generated using the Anisotropic Analytical Algorithm (AAA), our current institutional practice has transitioned to Acuros XB (AXB) for SRS planning. Although AXB offers improved accuracy in heterogeneous media,^37^, ^38^ intracranial SRS treatments are generally homogeneous in composition, with both tumor and surrounding brain tissue having similar densities. Prior study comparing AAA to AXB have shown minimal differences in dose calculations within homogeneous media.^39^ However, we acknowledge that AXB's more advanced scatter modeling may yield slightly different absolute values for dosimetric metrics, particularly for parameters like %D_1cm_, which are sensitive to peripheral dose gradients. The regression models developed in this study are based on relative relationships, and we anticipate that the overall trends and correlations would remain consistent regardless of the dose calculation algorithm. Nonetheless, future work may explore the robustness and transferability of these models across different dose calculation platforms to ensure broader clinical applicability.

## 5 CONCLUSION

This study confirms significant correlations between dosimetric parameters and PTV_Total_ in SRS planning using VMAT. These relationships were quantified using regression models, offering a data‐driven framework for predicting plan quality metrics based on tumor volume. Clinically, this approach enables greater consistency in plan evaluation, helps reduce inter‐institutional variability, and provides planners with tumor‐specific benchmarks for intermediate and low‐dose conformity.

## AUTHOR CONTRIBUTIONS

Shada Wadi‐Ramahi: Idea concept; Data collection; Statistical analysis; Discussion and Manuscript writing. Meysam Tavakoli: Data collection; Statistical analysis; Discussion and Manuscript writing. Serah Ashmeg: Data collection; Statistical analysis; Discussion and Manuscript review. Ron Lalonde: Idea concept; Discussion and Manuscript review. Zaid Siddiqui: Idea concept; Discussion and review. Shada Wadi‐Ramahi and Meysam Tavakoli are equally contributing first authors.

## CONFLICT OF INTEREST STATEMENT

Meysam Tavakoli, Sarah Ashmeg, Ron Lalonde, Zaid Siddiqui: None

Shada Wadi‐Ramahi: Associate Editor of JACMP.

## ETHICS STATEMENT

This work was carried under IRB approval number STUDY20070273.

## Data Availability

Research data are stored in an institutional repository and will be shared upon request to the corresponding author.
